# A controlled lumbar puncture procedure improves the safety of lumbar puncture

**DOI:** 10.3389/fnins.2023.1304150

**Published:** 2023-12-05

**Authors:** Chun Li, Miaomiao Li, Yixian Wang, Shaoyi Li, Lin Cong, Weining Ma

**Affiliations:** ^1^Department of Pediatrics, Shengjing Hospital of China Medical University, Shenyang, China; ^2^Department of Neurosurgery, Shengjing Hospital of China Medical University, Shenyang, China; ^3^Department of research and development, Medimicro (Tianjin) Medical Device Co., LTD, Tianjin, China; ^4^Department of Neurology, Shengjing Hospital of China Medical University, Shenyang, China

**Keywords:** lumbar puncture, high intercranial pressure, puncture needle, the CSF opening pressure, post-dural puncture headache

## Abstract

**Background:**

In order to improve the safety of lumbar puncture (LP), we designed a new type of LP needle, that is, an integrated and controlled LP needle, which can actively and accurately control the flow rate and retention of cerebrospinal fluid (CSF) during puncture, so as to achieve a controlled LP procedure.

**Objective:**

To evaluate whether a controlled LP procedure can improve the comfort of LP and reduce the risk of complications associated with LP.

**Methods:**

Patients requiring LP (n = 63) were pierced with an integrated and controlled LP needle or a conventional LP needle. The differences in vital signs, symptom score, comfort, operation time, CSF loss, CSF pressure fluctuation and back pain before and after puncture were analyzed.

**Results:**

An integrated and controlled LP needle (n = 35) significantly improved patients’ headache symptoms before and after puncture. In addition, a controlled LP procedure significantly reduced the amount of unnecessary CSF loss (*p* < 0.001), shortened the time of puncture (*p* < 0.001), improved patient comfort (*p* = 0.001) and reduced the incidence of back pain (*p* < 0.001). For patients with high intracranial pressure (HICP), the fluctuations in pressure of the CSF were also reduced while obtaining similar amounts of CSF (*p* = 0.009).

**Conclusion:**

A controlled LP procedure avoids unnecessary CSF loss, prevents rapid fluctuations in CSF pressure in patients with HICP, and reduces the risks associated with LP.

## Introduction

Lumbar puncture (LP) is a routine clinical diagnostic and treatment operation, used for the diagnosis of various inflammatory diseases of the central nervous system, cerebrospinal fluid (CSF) dynamic testing, drainage of intracranial high pressure and intrathecal injection (Wright et al., 2012). According to statistics, the number of LPs performed in hospitalized patients in the United States reached more than 360,000 in 2010 (Vickers et al., 2018). However, LPs may cause many complications, such as post-dural puncture headache (PDPH), and even cerebral hernia, which may endanger the safety of patients (Costerus et al., 2018). In recent years, the use of a atraumatic needle for LP has reduced PDPH due to CSF leakage caused by conventional needles to some extent (Nath et al., 2017, 2018), and has been recommended as the preferred needle type for an LP (Rochwerg et al., 2018). In terms of overall structure, both atraumatic and conventional needles are split structures composed of an external and an internal needle. Therefore, during the LP, the CSF naturally flows from the external needle passage due to intracranial pressure (ICP). When the patient has high intracranial pressure (HICP), the CSF outflow rate is faster. This may result in a large volume of outflow of CSF in a brief period of time. Due to the rapid outflow, the volume of CSF cannot be estimated, and the CSF opening pressure (OP) at this time is not accurate. In addition, a transient headache may occur after the puncture (Monserrate et al., 2015) and, in certain instances, the formation of secondary cerebral hernia due to the sudden change of the pressure gradient in the central nervous system (Wright et al., 2012). Therefore, how to improve the safety of LP, especially in patients with HICP, is a problem which needs to be addressed.

**Figure 1:**
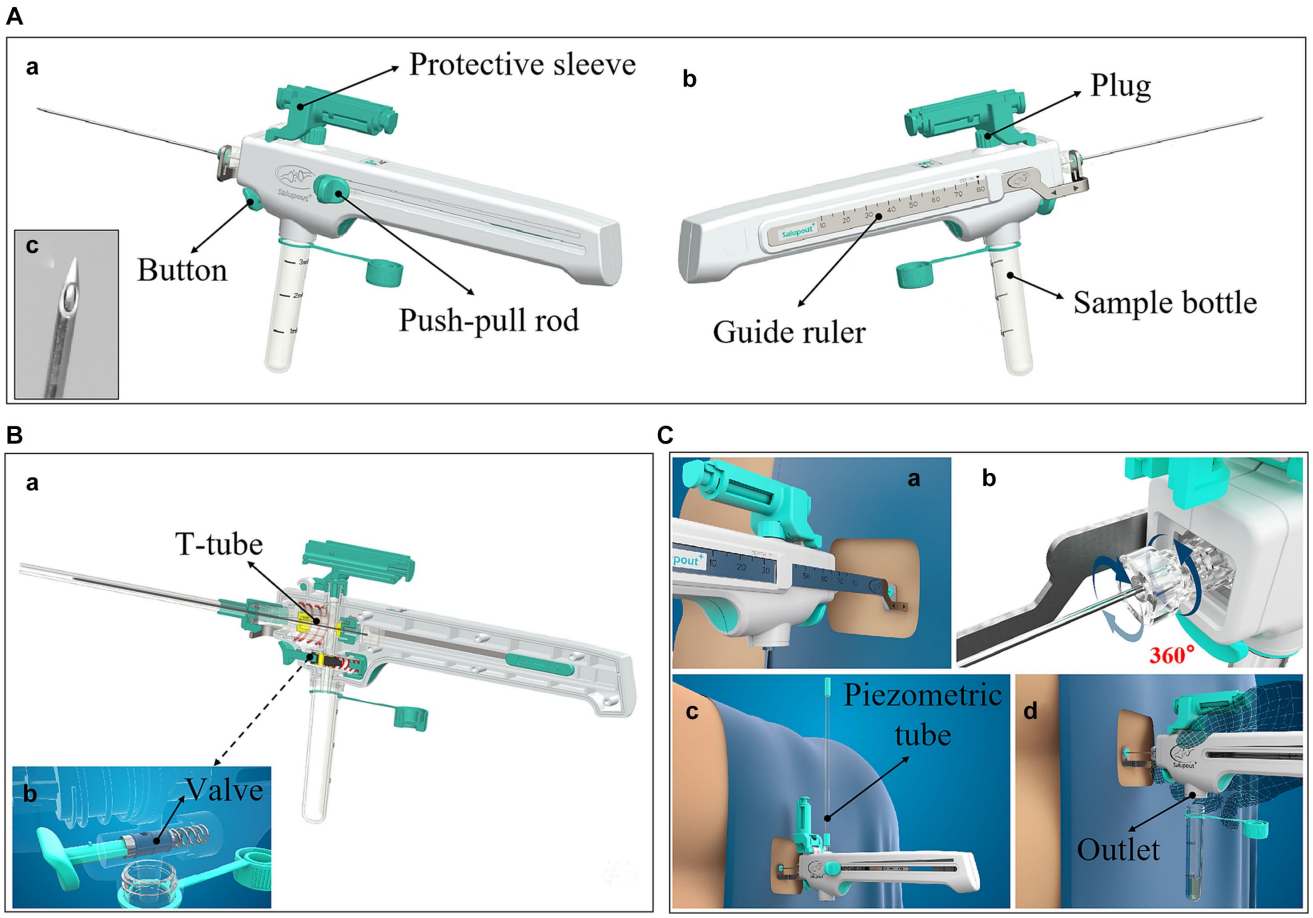
The details of the integrated and controlled LP needle. **(A)** Lateral view; **(a)** The left lateral view shows the push-pull rod, which connects the internal needle and moves it back and forth within the needle body; **(b)** The right lateral view shows the guide ruler, which not only stabilizes the external needle but also measures the puncture depth in real time; **(c)** The tip of the needle is atraumatic; **(B)** Cutaway view; **(a)** The cutaway view shows the internal mechanism of the needle, including the T-shaped pipeline, the pressure channel, the collection channel, the point where the internal needle is connected to the push-pull rod, and the valve; **(b)** This view shows how the valve works; **(C)** Functional display view of the needle; **(a)** The guide ruler measures the puncture depth in real time; **(b)** The external needle can be rotated 360 degrees; **(c)** The pressure channel is connected to a piezometric tube to measure the CSF pressure; **(d)** The outlet of the collection channel can be connected to the sample bottle to hold the CSF sample.

In this study, we conducted a controlled LP procedure using a newly designed integrated and controlled LP needle (Figure 1), and compared this with a conventional LP needle. The effects of the two on the vital signs and symptom scores before and after the puncture, and whether there were significant differences in comfort, operation time, CSF loss, CSF pressure fluctuations, and back pain were analyzed.

## Materials and methods

### Case selection

The enrolled patients were from the department of Neurosurgery and Neurology. Enrollment criteria: (1) age over 18 years old; (2) imaging examination such as head CT or MRI before LP; (3) indications for LP, such as obtaining CSF samples for testing, and CSF discharge test in patients with hydrocephalus. Exclusion criteria: (1) unstable vital signs; (2) definite cerebral hernia symptoms, such as bilateral anisoglyphic pupils; (3) definite infection focus at the puncture site. A total of 63 patients met the criteria. The enrolled patients provided written informed consent for the use of their data. This study was approved by the Ethics Committee of Shengjing Hospital of China Medical University.

### Research methods

**Figure 2:**
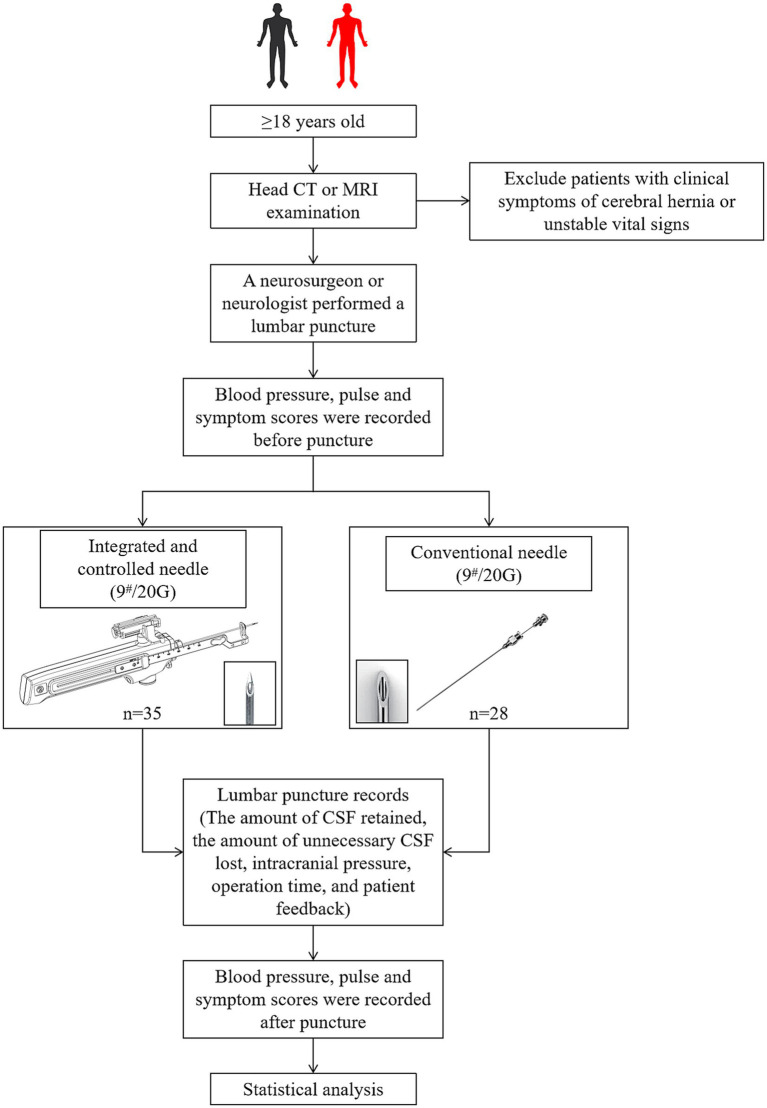
The design scheme and process of this study.

Patients meeting enrollment criteria were randomly assigned to the integrated and controlled LP needle group or the conventional LP needle group. LPs were performed by the same neurosurgeon in all neurosurgery enrolments, and likewise LPs were performed by the same neurologist in neurology enrolments (Figure 2). The LP procedure was recorded by the doctor: the subjects’ blood pressure, pulse rate and symptom score, including headache degree and accompanying symptom score, were recorded within 30 min before puncture, at the end of puncture, and 30 min, 2 h, and 4 h after puncture; The amount of CSF sample to be retained, CSF loss, CSF opening pressure (OP) (Hawker et al., 2011; Wang et al., 2019), CSF pressure after CSF sample collection, operation time and back pain, as well as comfort during puncture were recorded. After all eligible patients were enrolled, the study records were reviewed and statistically analyzed.

### Quantification of the study indicators

(1) Total symptom score: 0–4 points, which consisted of two parts: headache degree grade score (0–3 points) and concomitant symptom score (0–1 points). Among them, the headache degree score was graded according to the Numeric Rating Scale for Pain (NRS Pain) (Bø and Lundqvist, 2020), namely 0 points: NRS score 0; 1 point: NRS score 1–3; 2 points: NRS score 4–6; 3 points: NRS score 7–10; while for the presence of symptoms, 0 indicated no concomitant symptoms and 1 point indicated concomitant symptoms. (2) The index of intracranial hypertension: the CSF OP is not less than 200 mm H_2_O. (3) Calculation of the amount of CSF to be retained: the amount of sample in the sample bottle to be retained for subsequent laboratory examination. (4) Calculation of CSF loss: the amount of CSF accidentally lost during the operation, including dripping on the curved dish or the number of drops of CSF released (9#/20G: 20 drops equals 1 milliliter). (5) Calculation of CSF pressure fluctuations: CSF OP minus the CSF pressure value after taking the CSF sample. (6) Measurement of puncture depth: an integrated and controllable LP needle has a guide ruler on the side, and the puncture depth can be read through the scale on the guide ruler.

### LP method and the type of LP needle

**Figure 3:**
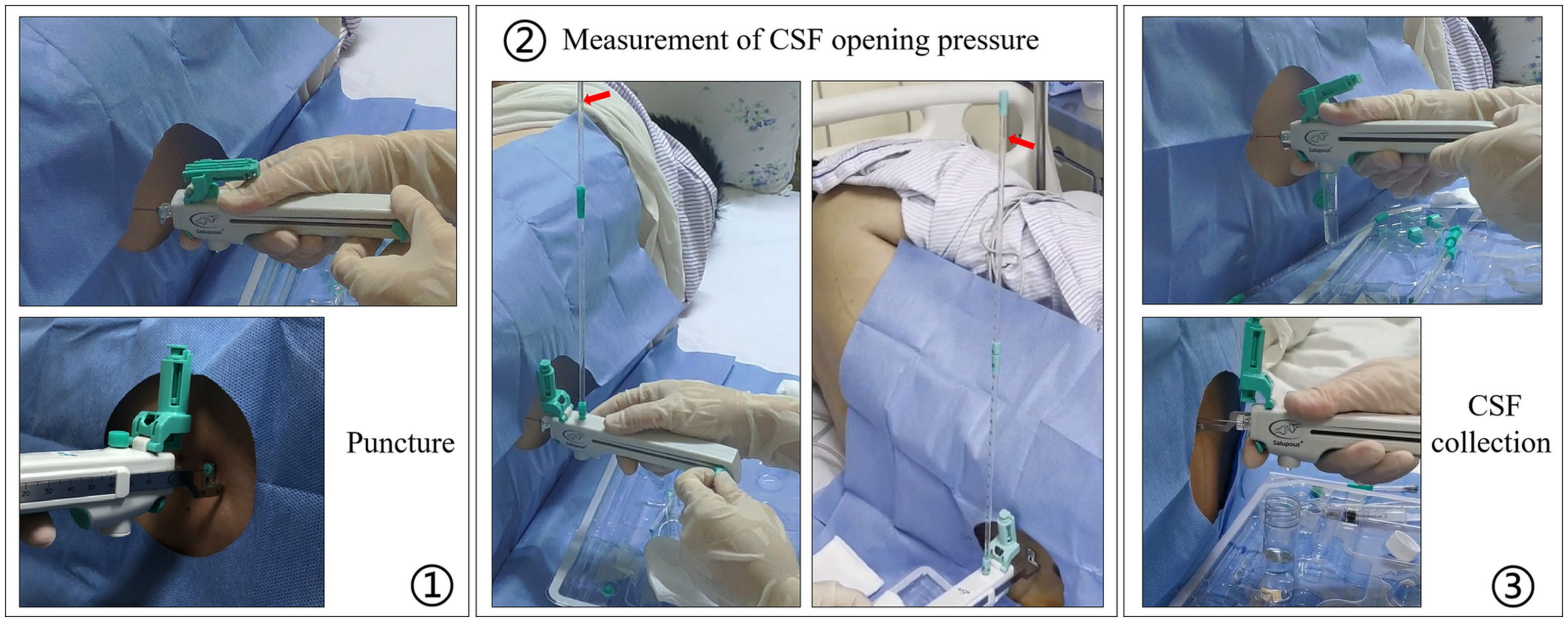
The controlled LP procedure with the integrated and controlled LP needle.

The patient was placed in the lateral decubitus position with his legs bent and his knees clasped. The tip of the integrated and controlled LP needle (9#/20G, Medimicro-Tianjin, China) is atraumatic. The controlled LP procedure was as follows (Figure 3): (1) the puncture needle and protective sleeve were removed and the guide ruler was pushed to the front end. A puncture was performed and it was confirmed that the needle entered the subarachnoid space. (2) Measurement of CSF OP: the plug at the outlet of the pressure channel was removed, the pressure tube connected, and the push-pull rod moved to the last site. After the test was completed, the push-pull rod was moved to the front to close the pressure channel, the pressure tube was removed, and the plug was tightened. (3) Collection of a CSF sample: the sample bottle was taken out and connected to the outlet of the collection channel, the push-pull rod moved to the last site, the button pressed to collect the required amount of CSF, and then released to stop the outflow of CSF. Finally the sample bottle was removed. After the procedure was completed, the push-pull rod was pushed to the front and the puncture needle was removed.

The tip of a conventional LP needle (9#/20G) is oblique. The LP procedure was as follows: (1) puncture was performed and entry of the needle into the subarachnoid space was confirmed (CSF outflow after pulling out the internal needle). (2) Measurement of CSF OP: after pulling out the internal needle, the glass pressure gage tube was connected to read the pressure value. (3) Collection of the CSF sample: after removing the pressure measuring tube, the sample bottle was connected to collect the required CSF sample, then the internal needle was returned, and the puncture needle was pulled out.

### Statistical methods

The paired sample T-test (two-tailed) was used to calculate statistical differences in blood pressure, pulse, operation time, CSF loss and CSF pressure fluctuation between the integrated and controlled LP needle and the conventional LP needle. The Chi-square test of independence was used to compare differences in the distribution of symptom scores, comfort, and the incidence of back pain. SPSS 17.0 software was used for statistical analysis, and *p < 0.05* was considered statistically significant.

## Results

### Case characteristics

LP was performed in 35 patients with an integrated and controlled LP needle and in 28 patients with a conventional LP needle. There was no significant difference in the age or gender distribution of the cases in the two groups (*p* > 0.05). The enrolled cases included inflammation, brain tumor, cerebrovascular disease, hydrocephalus, brain trauma, facial spasm, epilepsy and other neurological diseases (Table 1).

**Table 1:** Data of enrolled cases.

		Integrated and controlled needle	Conventional needle	*p*
Number of cases		35	28	
Age		53 ± 17	48 ± 15	0.252
Gender		Male: 20 Female: 15	Male: 11 Female: 17	0.159
Categories of neurological diseases	Inflammation	7	9	
	Brain tumor	9	8	
	Cerebrovascular disease	9	5	
	Hydrocephalus	6	1	
	Brain trauma	1		
	Facial spasm	3	2	
	Epilepsy		3	

The controlled LP process significantly improved patients’ headache symptoms.

**Figure 4:**
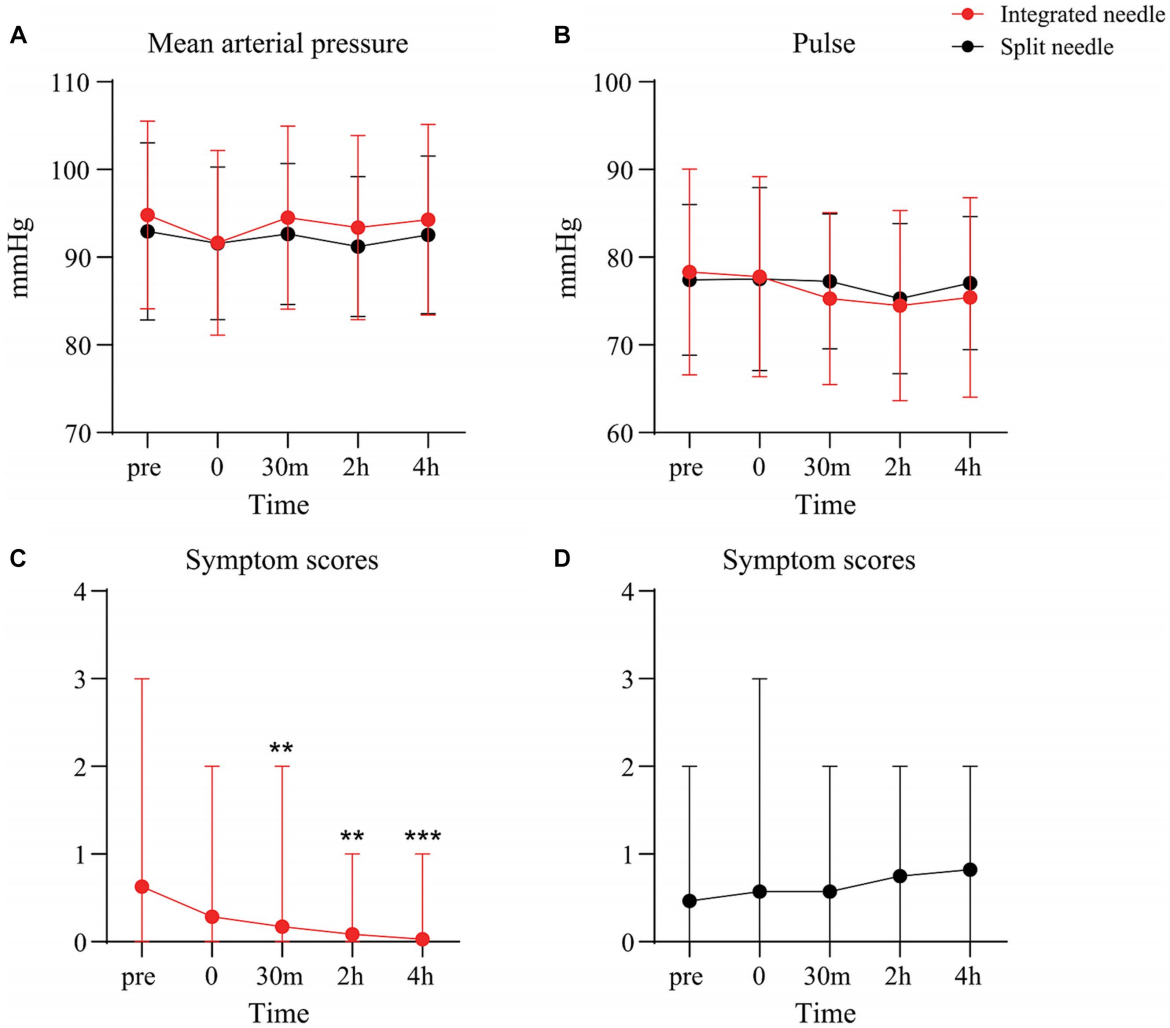
Changes of vital signs and symptom scores with the two types of puncture needle before and after puncture. **(A)** Mean arterial pressure curve; **(B)** Pulse curve; **(C)** Symptom score curve – integrated and controlled LP needle; **(D)** Symptom score curve – conventional LP needle.

Compared with LP performed with the conventional LP needle, the controlled LP procedure showed no significant difference in the impact on patients’ vital signs before and after puncture (*p* > 0.05) (Figures 4A,B). As shown in Figure 4C, patients undergoing LP with an integrated and controlled LP needle had significantly improved symptom scores at 30 min after puncture compared to before puncture (*p* < 0.01), and decreased thereafter, most significantly up to 4 h after puncture (*p* < 0.001). However, the symptom score curve of patients undergoing LP with a conventional LP needle increased (*p* > 0.05) (Figure 4D). Comparison of the difference in symptom score and number distribution between the two kinds of puncture needles before and after puncture showed that from 30 min after puncture, the proportion of patients with headache or headache aggravation in the conventional LP needle group gradually increased (*p* < 0.05) (Table 2).

**Table 2:** Case number distribution of symptom scores before and after puncture.

	Symptom scores										*p*
	Integrated and controlled needle (*n* = 35)					Conventional needle (*n* = 28)					
Time	0	1	2	3	4	0	1	2	3	4	
Pre	20	11	1	3	0	19	5	4	0	0	0.384
0	26	8	1	0	0	17	6	4	1	0	0.250
30 m	30	4	1	0	0	15	9	4	0	0	0.005
2 h	32	3	0	0	0	14	7	7	0	0	<0.001
4 h	34	1	0	0	0	13	7	8	0	0	<0.001

### The controlled LP procedure avoids the accidental loss of CSF and improves patient comfort

As shown in Table 3, compared with the conventional LP needle, application of the integrated and controlled LP needle for LP avoided additional loss of CSF (*p* < 0.001), shortened LP time (*p* < 0.001), and reduced the incidence of back pain after puncture (*p* < 0.001). In addition, the controlled LP procedure significantly improved patient comfort during the operation (*p* = 0.001), and the accurately measured puncture depth was also beneficial for subsequent operations.

**Table 3:** The difference between two needle types during lumbar puncture.

	Integrated and controlled needle	Conventional needle	*p*
Number of cases	35	28	
Operation time (min)	7.3 ± 2.1	11.9 ± 5.4	<0.001
The amount of CSF retained (mL)	8.7 ± 2.2	9.4 ± 1.8	0.191
Unnecessary loss of CSF (mL)	0.05 ± 0.01	2.1 ± 1.1	<0.001
CSF pressure fluctuations before and after puncture (mmH_2_O)	70.9 ± 37.1	71.6 ± 53.4	0.705
Puncture depth (cm)	5.4 ± 0.4	–	
Complaint of discomfort during puncture	0	9 (32.1%)	0.001
Back pain after puncture	1 (2.9%)	11 (39.3%)	<0.001

The controlled LP procedure reduced the fluctuation of CSF pressure during LP in patients with HICP, and improved the safety of LP.

**Figure 5:**
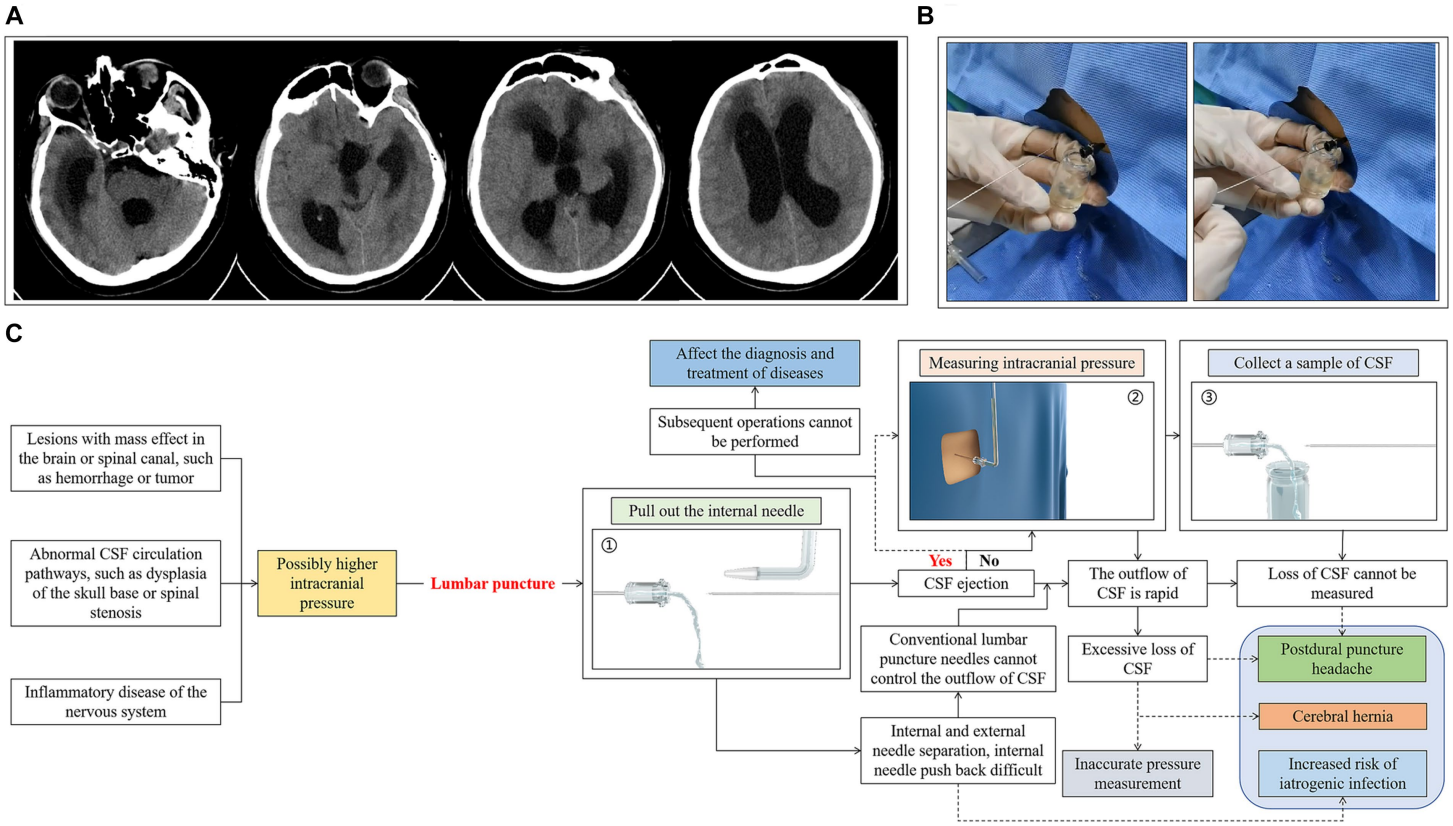
LP with a conventional LP needle in a patient with HICP. **(A)** Case one, male, 38 years old, who underwent resection of a third ventricle mass lesion. Two months after surgery, the patient presented with vomiting, disturbance of consciousness, and computed tomography (CT) examination suggested hydrocephalus. **(B)** CSF flow speed was fast, internal needle was contained in the needle cavity to control the flow speed of the cerebrospinal fluid. **(C)** Possible problems and related complications associated with use of the split LP needle in LP of patients with HICP.

**Figure 6:**
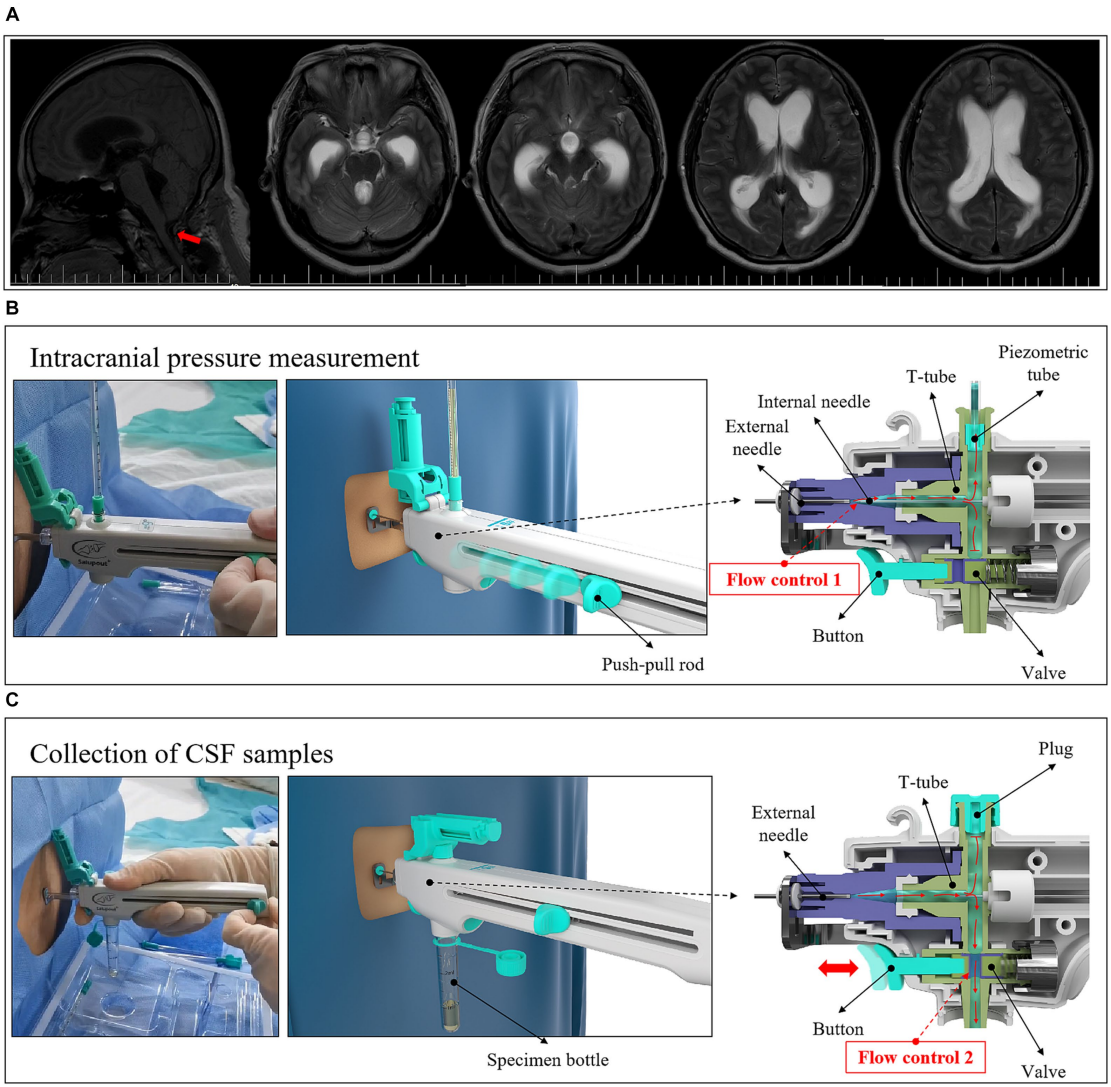
LP with an integrated and controlled LP needle in patients with HICP. **(A)** Case two, female, 47 years old, who underwent partial posterior cervical nerve root resection. One month after the operation, paroxysmal head pain began to appear, accompanied by nausea and vomiting, which was sometimes jet-like. Imaging examination suggested hydrocephalus. **(B)** The internal structure of the puncture needle and the principle of controlling CSF flow in the measurement of CSF pressure. **(C)** The internal structure of the puncture needle and the principle of controlling the flow of CSF during collection of a CSF sample.

The conventional LP needle could not easily control the flow of CSF during the LP procedure, especially in patients with HICP (Figures 5A,B), and the amount of unnecessary CSF loss was significantly increased compared with patients with normal intracranial pressure (ICP) (*p* < 0.001) (Table 4 and Figure 5C). However, when the integrated and controlled LP needle was used in patients with HICP (Figure 6A), the CSF flow process could be actively controlled through its internal structure. The integrated and controlled LP needle is still atraumatic but has the added benefits of pressure and collection channels, with the two connected together through a special “T-shaped pipeline.” The pressure and collection channels are used to control CSF flow during ICP measurement and CSF sample collection, respectively. In the pressure channel, the axes of the external and internal needles are at the same horizontal line, and the internal needle is moved horizontally forward and backward by a push-pull rod. The CSF outlet behind the external needle is “horn” shaped, and the gap between the internal needle and external needle outlet is used to control the flow of CSF (*Flow control 1*) (Figure 6B). Because the external needle and internal needle are always at the same level, the internal needle can quickly be retracted during the LP. In the collection channel, the release rate of CSF is controlled through the valve so that the amount of CSF outflow is precisely controlled. Specifically, the valve is controlled by a button. The pressure of the button changes the size of the outlet inside the valve, which determines the outflow rate of CSF (*Flow control 2*) (Figure 6C). Therefore, under the premise of retaining a similar amount of CSF sample, the controlled LP procedure can significantly reduce the fluctuation of CSF pressure in patients with HICP compared with the conventional LP needle procedure (*p* = 0.009). The incidence of headache aggravation immediately after puncture was also reduced (*p* = 0.02) (Table 4).

**Table 4:** Comparison of two puncture needles in patients with high cranial pressure.

	Integrated and controlled needle			Conventional needle			*p*
Number of cases	15			6			
Unnecessary loss of CSF (mL)	High cranial pressure	Normal cranial pressure	*P*	High cranial pressure	Normal cranial pressure	*p*	
	0.055 ± 0.02	0.05	0.284	3.83 ± 0.52	1.66 ± 0.73	<0.001	
CSF OP	244.5 ± 52.9			261.7 ± 55.3			0.546
The amount of CSF retained (mL)	9.1 ± 1.9			9.2 ± 0.8			0.888
Unnecessary loss of CSF (mL)	0.055 ± 0.02			3.83 ± 0.52			<0.001
CSF pressure fluctuations before and after puncture (mmH_2_O)	98 ± 33.2			150 ± 33.5			0.009
The headache worsened immediately after the puncture	0			4			0.02

## Discussion

### Principal findings

To the best of our knowledge, we have conducted the first study on the safety of LP using an integrated and controlled LP needle. During the puncture, we use the integrated and controlled LP needle to control the process of CSF flow, including velocity and direction, which can avoid unnecessary loss of CSF, reduce rapid fluctuations of ICP, and improve the safety of LP.

### Comparison with the literature

In this study, the proportion of headache after LP was higher in the conventional needle group, which was considered to be related to CSF leakage. Although the incidence of PDPH can be reduced by a small caliber atraumatic needle (Salzer et al., 2020; Hampel et al., 2022), due to its split structure, it is still difficult to control the flow of CSF during an LP, and it still results in excessive loss of CSF caused by HICP and the occurrence of related complications. As shown in this study, the amount of CSF lost during LP was significantly higher in patients with HICP than in patients with normal ICP, and CSF pressure fluctuated significantly. Moreover, the proportion of patients suffering headache aggravation immediately after puncture was higher. In clinical practice, it is well known that the ICP of the patient is often unknown before an LP, even though imaging is available. When a split LP needle is used for LP, if cases of HICP are encountered, CSF ejection may occur after the needle enters the subarachnoid space and the internal needle is pulled out. Excessive outflow of CSF in a brief period of time may result in rapid changes in the brain–spinal cord pressure gradient, or even cause cerebral hernia (Durand et al., 1993; Brouwer et al., 2014), endangering the patient’s life. In general, doctors usually need to rely on personal experience to complete the entire LP procedure. For example, CSF pressure can be judged by the outflow velocity of the CSF or the impact force of CSF on the finger, which requires abandonment of the CSF pressure testing process; temporary rapid infusion of mannitol during operation to reduce ICP; or indwelling of the internal needle within the needle cavity to control the outflow rate of CSF. However, sometimes the ICP is so high that the CSF pressure test and the retention of CSF samples are extremely difficult, which interferes with subsequent treatment of the patient. Therefore, to perform a safe LP on patients, it is important not only to evaluate imaging data prior to the puncture (van Crevel et al., 2002) but also to control the flow of CSF during the puncture. In this study, a controlled LP procedure was achieved by applying an integrated and controlled LP needle. During the LP procedure, the CSF was locked in the puncture needle, and the flow direction, speed and collection amount of CSF were accurately controlled by the doctor during the CSF pressure test and CSF sample retention steps, to avoid large fluctuations of CSF pressure. This improved patient comfort during operation and reduced the risk of cerebral hernia. In addition, the incidence of headache and back pain after puncture was also low, which may also be related to use of the atraumatic type of integrated and controlled LP needle (Costerus et al., 2018).

In this study, patients with hydrocephalus underwent CSF OP testing before shunt surgery. Previous studies have shown that the shunt valve should be set at a pressure like the CSF OP measured during LP to avoid excessive shunting after surgery (Khan et al., 2013; Vivas-Buitrago et al., 2020). It has been reported that an atraumatic spinal needle is beneficial to recording more accurate CSF OP (Woo et al., 2022). However, when using the split LP needle, the internal needle needs to be pulled out before connecting the measuring tube to measure the CSF OP, and some of the CSF is inevitably lost during the process. In contrast, the integrated and controlled LP needle has almost no loss of CSF before the CSF OP measurement. Thus, measurement of the CSF OP may be more accurate and may be a more effective guide to shunt valve pressure setting. Of course, further research is needed to compare the accuracy of the two puncture needles in measuring the OP of CSF.

### Limitations

One limitation is that the use of the integrated and controlled LP needle is complex than the conventional needle, and the change in operating habits requires a certain learning process. From our experience, when familiar with the use of the method, the operation is more stable and comfortable. Another limitation is that the proportion of HICP cases is relatively small. We will further study how to further improve the safety of LP in cases with HICP.

### Next steps

This involves the further improvement of the integrated and controlled LP needle, such as the application of pressure sensors to measure the CSF pressure during the puncture procedure in real time, which can not only further reduce the unnecessary loss of CSF, but also improve the accuracy of CSF pressure measurement. In addition, the miniaturized ultrasonic probe is integrated into the front end of the guide ruler of the needle, which can guide the puncture direction while monitoring the puncture depth and improve the success rate of LP.

## Conclusion

The integrated and controlled LP needle controlled the flow of CSF during an LP and avoided rapid loss of CSF in a brief period of time. In addition, the CSF sequestered in the puncture needle, and the outflow rate of CSF, was controlled by the doctor, which avoided rapid changes of CSF pressure during the puncture process in patients with HICP, and further reduced the possibility of cerebral hernia and other serious complications. A controlled LP procedure is beneficial to reduce the incidence of complications associated with LP and improve the safety of LP.

## Data availability statement

The original contributions presented in the study are included in the article/supplementary material, further inquiries can be directed to the corresponding authors.

## Ethics statement

The studies involving humans were approved by the Ethics Committee of Shengjing Hospital of China Medical University (Ethical number: 2023PS771K). The studies were conducted in accordance with the local legislation and institutional requirements. The participants provided their written informed consent to participate in this study.

## Author contributions

CL: Conceptualization, Formal analysis, Writing – original draft. ML: Data curation, Writing – original draft. YW: Methodology, Writing – review & editing. SL: Project administration, Supervision, Writing – review & editing. LC: Supervision, Validation, Writing – review & editing. WM: Conceptualization, Formal analysis, Investigation, Methodology, Validation, Writing – review & editing.
